# Differential Expression of Cholecystokinin A Receptor in Gallbladder Cancer in the Young and Elderly Suggests Two Subsets of the Same Disease?

**DOI:** 10.1155/2014/625695

**Published:** 2014-06-15

**Authors:** Hasan Raza Kazmi, Abhijit Chandra, Kavita Baghel, Anshuman Singh, Jaya Nigam, Devendra Parmar, Abbas Ali Mahdi, Sudhir K. Goel, Sandeep Kumar

**Affiliations:** ^1^Department of Surgical Gastroenterology, King George's Medical University, Lucknow 226003, India; ^2^Developmental Toxicology Division, Indian Institute of Toxicology Research, Lucknow 226003, India; ^3^Department of Biochemistry, King George's Medical University, Lucknow 226003, India; ^4^Department of Biochemistry, All India Institute of Medical Sciences, Bhopal, Madhya Pradesh 462024, India; ^5^All India Institute of Medical Sciences, Bhopal, Madhya Pradesh 462024, India

## Abstract

*Background*. Cholecystokinin type A receptor (CCKAR) is known to be overexpressed in variety of human malignancies but information regarding its expression in gallbladder cancer (GBC) is limited. Attempts were now made to investigate expression pattern of CCKAR mRNA and protein in controls and GBC patients and correlate it with various clinicopathological parameters following surgical resection. *Materials and Methods*. Gallbladder tissue samples from 64 subjects (GBC: 39; control: 25) were studied. Expression of CCKAR mRNA was evaluated by reverse transcriptase-polymerase chain reaction and confirmed using real-time polymerase chain reaction. Protein expression was studied by enzyme-linked immunosorbent assay. *Results*. Significantly higher expression of CCKAR mRNA (*P* < 0.0001) and protein (*P* < 0.0001) was observed in GBC tissues. Overexpression was also observed for stage III and in moderately and poorly differentiated tumors. When the clinicopathological parameters were compared, we found age dependent decrease in CCKAR expression. Relatively higher expression of CCKAR was observed in younger patients (age < 45 years) having more aggressive disease when compared with elderly ones (age ≥ 45 years). *Conclusions*. Age related differential expression of CCKAR in GBC may suggest two possible variants of the disease in this endemic belt.

## 1. Introduction

Gallbladder cancer (GBC) is the most common malignancy of the biliary tract with high incidence in Chile, Japan, and Northern India (incidence rate of 7.4 per 10^5^ for females and 3.6 per 10^5^ for males in Delhi) [1–3]. Complete surgical resection remains the only potential curative treatment for this malignancy but with high recurrence rate. However, at the time of diagnosis most patients have unresectable disease and only about 20% of patients are suitable candidates for surgery [4, 5]. Further, the 5-year survival rate is just 15% for the resected cases [6, 7].

Approach of* in vivo* targeting of human cancers through peptide receptors is gaining interest and hence screening novel therapeutic proteins remains subject of intense investigation [8]. Cholecystokinin (CCK) is a gastrointestinal peptidyl hormone which stimulates pancreatic exocrine secretion, gut motility, and gallbladder contraction. Action of CCK on the gallbladder is mediated by high affinity seven-transmembrane spanning CCK type A receptor (CCKAR) belonging to G protein-coupled receptor's family [9, 10]. Decrease in CCK receptors may be involved in the pathogenesis of gallstone formation resulting in decreased gallbladder motility. It has been observed that gallstones are present in nearly 80% of patients with GBC [11, 12]. Moreover, abnormal processing of CCKAR is found to be associated with gallstones and obesity [13]. Expression of CCKAR has also been detected in various human cancers, including pancreatic malignancy [14–16]. However, information regarding its expression in GBC is limited. We aimed to study the expression of CCKAR in gallbladder malignancy and correlate it with the clinical presentation, including association with stones, stage of disease, and outcomes.

## 2. Materials and Methods

### 2.1. Clinical Samples

The ethics committee of the institute approved the protocol, and informed consent was obtained from each patient before enrolment. All procedures were in accordance with the Declaration of Helsinki. Thirty-nine resected GBC tissue samples were obtained through extended cholecystectomy between May 2010 and August 2012. Only resected GBC patients confirmed by postoperative biopsy were included in the study. Staging was done according to the American Joint Committee on Cancer tumor node metastasis classification (TNM), 2010 [17]. Patients not amenable for a curative resection (with advanced malignancy) were excluded. Twenty-five normal gallbladder tissue specimens obtained through surgery (removed as part of choledochal cyst excision (*n* = 12), Whipple's pancreaticoduodenectomy (*n* = 9), or following hepatobiliary trauma (*n* = 4)) were selected as controls. All control gallbladders were histopathologically normal. Although these tissues are referred to as normal gallbladder tissues, it is important to point out that they cannot be regarded as healthy normal specimens. However, this was the best possible way that normal control gallbladder could be obtained.

Tissue samples were taken in TRIzol (Invitrogen) and stored at −80°C until used for analysis. RNA was isolated as per the protocol of the supplier and its purity was checked by running it in 1% agarose gel. Total RNA (2 *μ*g) was transcribed to cDNA using High-Capacity cDNA Reverse Transcription Kit (Applied Biosystems, USA).

### 2.2. Reverse Transcription-Polymerase Chain Reaction (RT-PCR) and Gel Electrophoresis

Table 1 represents primer sequences that were used for amplification. Normalization was carried out using housekeeping gene, beta actin. The PCR cycling conditions (for CCKAR) were of initial denaturation of 5 min at 94°C, followed by 35 cycles at 94°C for 1 min and 60°C for 1 min and 72°C for 1 min and 30 s, and final extension of 72°C for 10 min. For beta actin, the PCR cycle conditions were of initial denaturation at 94°C for 5 min, followed by 30 cycles at 94°C for 30 s, 58°C for 30 s, and 72°C for 45 s, and final extension of 72°C for 10 minutes. Gene Amp PCR system 9700 (Applied Biosystems, USA) was used for amplification and products were analysed in 2% agarose gel stained with ethidium bromide in VERSA DOC Imaging system, Model 1000 (Biorad, USA). Densitometric analysis of the PCR products was done using Quantity One Quantitation Software version 4.3.1 (Biorad, USA).

### 2.3. Real-Time PCR Analysis

Real-time PCR assay reaction was conducted using 26 Power SYBR Green PCR master mix (Applied Biosystems, USA) as described earlier by Baghel et al. [24]. Through the use of human CCKAR sequences obtained from the GenBank database, primer sets were designed by using the software primer express 3.0 (ABI, USA). After comparison, the potential primer sets were identified. The specificity of the primers was then validated using sequencing. Melting curve profile obtained using the dissociation software of the real-time PCR apparatus also validated the specificity of the primer design. For each sample, PCR reaction was performed in triplicate. 7900HT Sequence Detector System software version 2.2.1 (Applied Biosystems, USA) was used to analyze the data.

### 2.4. ELISA

Expression of CCKAR protein was determined quantitatively using CCKAR antibody (Santa Cruz, USA). Initially, standard curve was plotted with known concentrations of antigen (0.312 ng/mg–60 ng/mg). In brief, 96-well immunoassay plates were coated with 100 *μ*L/well of diluted antigen for 2 hours followed by blocking step with blocking solution (1% BSA). Plates were incubated overnight with 100 *μ*L/well of diluted (1 : 1000) anti-human CCKAR antibody at 4°C. After washing away any unbound antibody, 100 *μ*L/well of secondary antibody (1 : 2000) was added, and incubation was done for 3 hours. This was followed by dispensation of 100 *μ*L/well of substrate solution (Super Signal ELISA Pico, Thermo Scientific). The enzyme-substrate reaction was stopped after sufficient colour development by adding 50 *μ*L/well of 0.5 M H_2_SO_4_. Colour change was measured spectrophotometrically at a wavelength of 450 nm.

### 2.5. Statistical Analysis

Statistical analysis was carried out using unpaired Student's* t*-test. *P* < 0.05 (two-tailed) was considered to be statistically significant. Correlation analysis was performed using Pearson correlation method. Clinical data was analyzed using Fisher exact or Chi-square test.

## 3. Results

**Figure 1:**
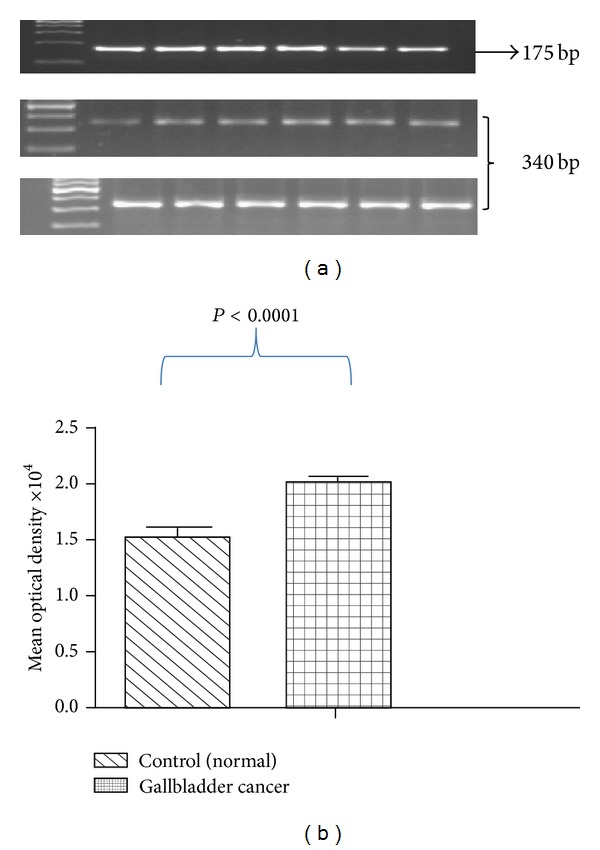
(a) Reverse transcription-polymerase chain reaction products of CCKAR mRNA in control gallbladder (middle row) and gallbladder cancer (lower row) tissues after normalization with housekeeping gene beta actin (upper row). DNA ladder is of 100 bp. (b) Graphical representation of mean band density of CCKAR PCR product in control gallbladder and gallbladder cancer. Data is represented as mean ± standard error. CCKAR: cholecystokinin type A receptor and PCR: polymerase chain reaction.

**Figure 2:**
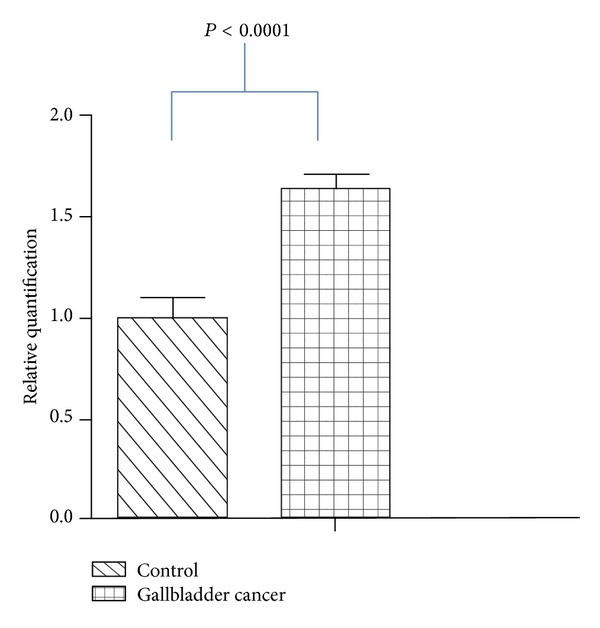
Graphical representation of CCKAR mRNA expression in gallbladder cancer tissues by real-time PCR. All the values are mean ± standard error. CCKAR: cholecystokinin type A receptor and PCR: polymerase chain reaction.

Characteristic profiles of subjects enrolled in the study are shown in Table 2. Significant increase (23.27%, *P* < 0.0001) of CCKAR mRNA in GBC tissues was observed as compared with controls. The band size for CCKAR mRNA was 375 bp and that of beta actin was 175 bp (Figure 1). Further, stratification of band intensity in relation to the stage of tumor revealed significant increase (13.23%, *P* < 0.0001) in expression of CCKAR mRNA in stage III as compared with stage II GBC. Quantitative real-time PCR assay also revealed significantly higher (63.67%, *P* < 0.0001) expression of CCKAR mRNA in GBC tissues (Figure 2). Higher expression (26.94%, *P* < 0.0001) of CCKAR mRNA was observed in stage III as compared with stage II tumor. Increased expression (22.83%, *P* < 0.001) of CCKAR mRNA was observed for moderately and poorly differentiated tissues as compared with well-differentiated ones. However, no significant difference in expression was found for presence or absence of gallstones (*P* = 0.98).

**Figure 3:**
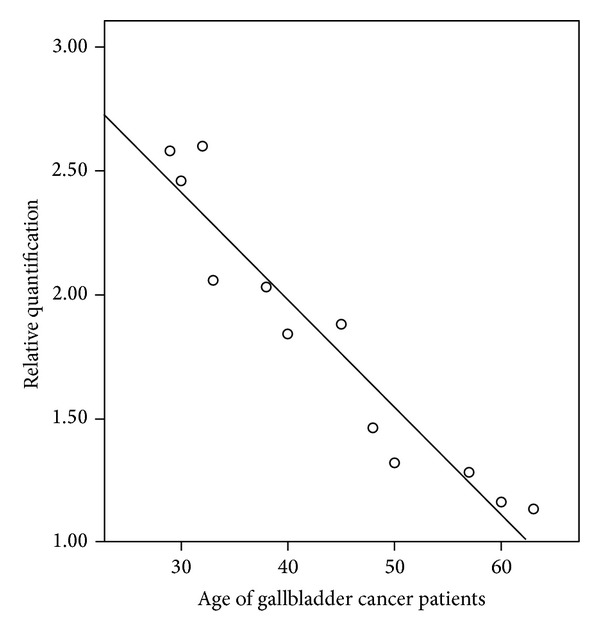
Scatter diagram showing correlation between relative quantification of cholecystokinin A receptor mRNA expression and age of gallbladder cancer patients. Correlation is significant at 0.01 level (2-tailed) with Pearson correlation coefficient, *r* = −0.956.

**Figure 4:**
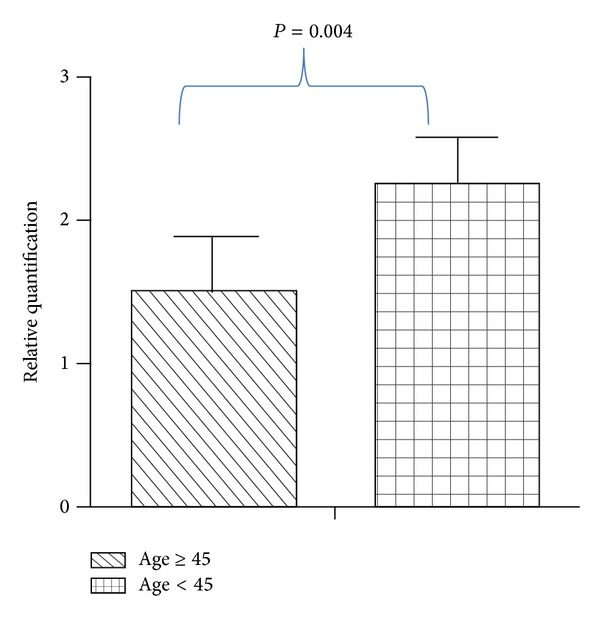
Comparison of relative quantification of cholecystokinin A receptor mRNA expression between age ≥ 45-year (*n* = 6) and age < 45-year (*n* = 6) human gallbladder cancer patients.

Significant correlation between CCKAR mRNA expression and age of GBC patients was observed, when relative quantification (by real-time PCR) was correlated with various clinicopathological parameters. We selected 12 GBC patients and 12 age and sex matched controls (for normalization in real-time PCR) from our enrolled patients and an age dependent decrease in the CCKAR mRNA expression was observed. The correlation was significant at 0.01 level (2-tailed) with Pearson correlation coefficient, *r* = −0.956 (Figure 3). As there was a marked decrease in the relative quantification after 45 years of age, we classified the GBC patient pool into two groups (A = age < 45 years (*n* = 6); B = age ≥ 45 years (*n* = 6)). Significantly higher expression (33.63%, *P* = 0.004) of CCKAR mRNA was observed in group A as compared to B (Figure 4). There were 17 GBC patients below age of 45 years while the rest (*n* = 22) of recruited subjects were above or equal to the age of 45 years. Tables 3 and 4 represent age-wise distribution of clinical presentation and histopathological parameters, respectively, for all GBC patients. The mean survival was significantly shorter in GBC patients with age < 45 years as compared to patients with age ≥ 45 years. The distribution of stage of tumor was found to be insignificant (*P* = 0.11) between age ≥ 45 years and age < 45 years while significant difference (*P* = 0.01) was observed for cellular differentiation between both groups.

At translational level, mean CCKAR protein concentrations were significantly higher (*P* < 0.0001) in GBC patients as compared to controls. Significantly higher (*P* = 0.03) CCKAR protein content was observed for stage III as compared with stage II GBC and for poorly and moderately differentiated tumors as compared with well-differentiated ones (*P* = 0.03). We observed significant increase (*P* < 0.0001) of mean CCKAR protein content in GBC patients with age < 45 years as compared with age ≥ 45 years. However, there was no significant difference in CCKAR protein content for presence or absence of gallstones for GBC patients (*P* = 0.92).

## 4. Discussion

CCK is an important gut hormone which regulates growth of various gastrointestinal malignancies along with normal tissues [25]. CCK receptors in the normal human gallbladder have been studied [26–28], and its expression has also been reported in various human malignancies (Table 5). Due to selective expression, CCKAR may serve as potential biomarker for pancreatic adenocarcinoma [21]. However, in GBC CCKAR mRNA and protein expression has not been extensively studied. CCKAR is known to be involved in the main pathway for gallbladder contraction and association of gallstones with GBC is also known [1, 27]. In a recent study, expression profile of CCKAR in GBC and gallstone disease by immunohistochemistry and immunoblotting was investigated, but its correlation with normal gallbladder was not studied [23]. In the present study, we analyzed CCKAR mRNA and protein expression in normal (control) gallbladder and GBC tissues. To the best of our knowledge, this is the first study evaluating expression of CCKAR at both transcriptional and translational levels in resected GBC.

Various studies reported the mean age of GBC presentation around 65 years with female preponderance [29–31]. However, studies from the Indian subcontinent show mean age to be 55 years [23, 32]. In our study, the mean age of GBC patients was 43.87 years with male to female ratio of 1 : 2.87. The early presentation in this endemic area is maybe due to early exposure to the risk factors.

Overexpression of CCKAR mRNA and protein in GBC tissues is the most relevant finding of this study. A significant age dependent decrease in CCKAR expression became evident with younger patients (age < 45 years) having higher expression of CCKAR as compared with elderly ones (age ≥ 45 years). Our data also showed that patients in the younger age presented more frequently with poorly and moderately differentiated tumors suggesting more advanced presentation of the disease. Survival after surgical resection of the younger GBC patients was found to be significantly less as compared with the elderly. The difference in clinical presentation along with differential CCKAR expression in GBC patients suggests two variants of the disease in this endemic belt with younger patients (age < 45 years) having more aggressive disease, poor surgical outcomes, and higher CCKAR expression in comparison to elderly group (age ≥ 45 years) having more indolent disease, better outcomes, and decreased CCKAR expression. Differential expression of CCKAR mRNA was also observed for tumor stage and differentiation. Rai et al. [23] found insignificant difference in the expression of CCKAR protein between various grades of tumor. This difference may be attributed to the method of detecting protein expression in both studies.

Limited information is available regarding expression of CCKAR protein in malignant tissues [20, 22, 23]. Most of the studies carried out so far were based on semiquantitative technique of immunohistochemistry or immunoblotting [22, 23]. In the present study, overexpression of CCKAR was observed in GBC indicating similar influence of CCK and its receptor in origin and growth of gallbladder malignancy. Our current findings are in line with the results of a previous study done so far [23]. Schaffer et al. [33] observed that multiple naturally occurring amino acid polymorphisms and/or mutations in transmembrane domain of CCK receptor in* Mastomys natalensis* may together result in ligand-independent CCK receptor overactivity which may lead to the development of tumor. Takata et al. [34] demonstrated that region downstream of −622 in the promoter region might regulate human CCKAR transcription. Differential expression of this receptor may be due to polymorphisms or mutation in the promoter or coding region which correlates with gene expression in the human gallbladder and needs further evaluation [34, 35].

We studied the expression of CCKAR mRNA and protein in resected stages II and III GBC tissues. In our recruited GBC patients, none were of stage I, as such cases are usually detected incidentally during cholecystectomy performed for benign diseases. Additional stagewise studies are required along with dysplasia samples to establish its role in gallbladder carcinogenesis. Also, investigation of age dependent change of CCKAR expression needs to be explored in much larger samples along with multivariable analysis and may be the area of future research.

Due to overexpression in many primary human cancers, peptides and peptide receptors remain an interesting candidate for treating cancers through receptor targeting approach [36]. Large body of data exists concerningseveral peptide and nonpeptidyl CCKAR modulators, and clinical potentials of these agents are under trial [37]. Our study defines two possible variants of GBC in this endemic belt. It also forms the basis for developing newer therapeutic options based on the CCKAR active drugs to obtain better outcomes following surgery in these variants.

## 5. Conclusions

We conclude that younger patients of gallbladder malignancy with higher expression of CCKAR have more aggressive disease and short survival as compared with elder ones. This differential expression of CCKAR reflects two subsets of GBC in North Indian population which needs to be evaluated further using larger sample size.

## Figures and Tables

**Table 1:** Primer sequences used for amplification of CCKAR and beta actin in RT-PCR and real-time PCR. CCKAR: cholecystokinin type A receptor and RT-PCR: reverse transcription-polymerase chain reaction.

Gene	Technique	Primer sequence	First author
CCKKAR	RT-PCR	Forward 5′ CCTACGACACCGCCTCCGC 3′ Reverse 5′ TCCGTTCTTTCTTCTCTGCCTCCT 3′	Mandair [18]
Beta actin	Real-time PCR	Forward 5′ CCTGGCACCCAGCACAAT 3′ Reverse 5′ GCCGATCCACACGGAGTACT 3′	—
CCKKAR	Real-time PCR	Forward 5′ GCGATTTGCAAACCCTTACAG 3′ Reverse 5′ CACCTTCAAAGCATGGGATTTT 3′	

**Table 2:** Characteristics of controls and gallbladder cancer (GBC) patients.

Parameters	Normal, *n* (%)	GBC, *n* (%)
Total	**25**	**39**
Male	11 (44)	12 (30.77)
Female	14 (56)	27 (69.23)
Mean age (years) ± S.D.	41.24 ± 13.27	43.87 ± 12.39
Range	19–63	21–65
Presence of gallstones	0	28 (71.79)
Stage	N/A^a^	
II		22 (56.41)
III		17 (43.59)
Tumor differentiation	N/A^a^	
Poorly and moderately		20 (51.28)
Well		19 (48.72)

^a^N/A: not applicable.

**Table 3:** Table showing clinical presentation of GBC patient for age < 45 years (*n* = 17) and age ≥ 45 years (*n* = 22).

Parameters	Age < 45 years (*n* = 17) *n* (%)	Age ≥ 45 years (*n* = 22) *n* (%)	*P* value
Abdominal pain	16 (94.12)	19 (86.36)	0.62
Weight loss	17 (100)	20 (90.91)	0.49
Palpable lump	9 (52.94)	3 (13.64)	0.01∗
Presence of gallstones	14 (82.35)	14 (63.64)	0.29
Survival in months ± S.D.	10.3 ± 3.18	14.08 ± 5.09	0.034∗

Values are given as numbers. Differences were tested by using Fisher exact test. ∗Indicates statistically significant difference (*P* < 0.05). GBC: gallbladder cancer.

**Table 4:** Comparison of histopathological parameters and tumor stage of gallbladder cancer patients between age < 45 years (*n* = 17) and age ≥ 45 years (*n* = 22).

Parameters	Age < 45 years(*n* = 17) *n* (%)	Age ≥ 45 years (*n* = 22) *n* (%)	*P* value
Histopathology			
Well-differentiated tumors	4 (23.53)	15 (68.18)	0.01∗
Poorly and moderately differentiated tumors	13 (76.47)	7 (31.82)	
Tumor stage			
Stage II	7 (36.36)	15 (65)	0.11
Stage III	10 (63.64)	7 (35)	

Values are given as numbers. Differences were tested by using Fisher exact test. ∗Indicates statistically significant difference (*P* < 0.05).

**Table 5:** CCKAR expression profile in human malignancies: literature review. CCKAR: cholecystokinin type A receptor.

First author	Tumour types	Number of samples	Methodology	Findings
Okada [19]	Gastric	14	RT-PCR	Suggest a greater role for CCK and CCKAR than for gastrin and CCK-B receptor in gastric cancers

Clerc [15]	Oesophageal, gastric, and colon cancer	8: oesophageal 12: colon 8: gastric	RT-PCR	The expression of CCKAR may be an important indicator of the influence of CCK on the origin and growth of these cancers

Reubi [20]	Various human malignancies	32: gastroenteropancreatic tumour 24: medullary thyroid carcinoma 16: neuroblastoma 27: meningioma 65: breast carcinoma	Receptor autoradiography	CCKAR rarely expressed in tumors except gastroenteropancreatic tumors (38%), meningiomas (30%), and some neuroblastomas (19%)

Weinberg [21]	Pancreatic cancer	22	RT-PCR	Overexpression of CCKAR mRNA in pancreatic cancer

Moonka [16]	Pancreatic cancer	30	RT-PCR	Increased expression of CCKAR mRNA may stimulate pancreatic cancer

Schulz [22]	Various human tumours	5: colorectal 5: pancreatic adenocarcinoma 5: breast 10: ovarian 4: prostate 6: thyroid 15: carcinoid 8: pancreatic insulinoma 4: pituitary adenoma 4: pheochromocytoma 4: glioblastoma 4: meningioma	IHC	CCKAR overexpression in a subset of human neuroendocrine tumours may provide a molecular basis for efficient targeting of these tumors with radiolabeled CCK analogs

Rai [23]	Gallbladder cancer	94	IHC	Significant increase in expression of CCKAR in gallbladder cancer as compared to gallstone disease

Current study	Gallbladder cancer	31 resected samples	RT-PCR, real-time PCR, and ELISA	Overexpression of CCKAR mRNA and protein in GBC tissues as compared with normal gallbladder suggests its therapeutic potential

RT-PCR: reverse transcriptase-polymerase chain reaction and IHC: immunohistochemistry.
